# Correction: Li et al. Extraction of Polysaccharides from Root of *Pseudostellaria heterophylla* (Miq.) Pax. and the Effects of Ultrasound Treatment on Its Properties and Antioxidant and Immune Activities. *Molecules* 2024, *29*, 142

**DOI:** 10.3390/molecules30051174

**Published:** 2025-03-06

**Authors:** Hangyu Li, Ziwei Liu, Qianqian Liu, Xinnan Zhang, Sheng Li, Feng Tang, Linzi Zhang, Qian Yang, Qiran Wang, Shuyao Yang, Ling Huang, Yuwei Ba, Xihui Du, Falong Yang, Haibo Feng

**Affiliations:** 1 College of Animal Husbandry and Veterinary Medicine, Southwest Minzu University, Chengdu 610041, China; li1998hangyu@163.com (H.L.); z774513726@126.com (Z.L.); 15892603728@163.com (Q.L.); n1547187363@163.com (X.Z.); listen699@163.com (S.L.); tangfeng1719556170@163.com (F.T.); zlz754130837@163.com (L.Z.); qianq1103@163.com (Q.Y.); w2329839437@163.com (Q.W.); carry_young@163.com (S.Y.); hl17723661622@163.com (L.H.); bayuwei1998@163.com (Y.B.); duxihui2022@163.com (X.D.); 2 Key Laboratory of Ministry of Education and Sichuan Province for Qinghai–Tibetan Plateau Animal Genetic Resource Reservation and Utilization, Chengdu 610041, China

**Errors in Figure**

Upon reviewing our published work [1], we realized that we mistakenly used incorrect figures (Merge-OVA+PHP2 in Figure 4F). To rectify this error, we have prepared the correct version of this image as featured in Figure 4 below. We understand the significance of maintaining the integrity and accuracy of scientific publications, and we are fully committed to ensuring the correctness of our work.

The authors state that the scientific conclusions are unaffected. This correction was approved by the Academic Editor. The original publication has also been updated.

## Figures and Tables

**Figure 4:**
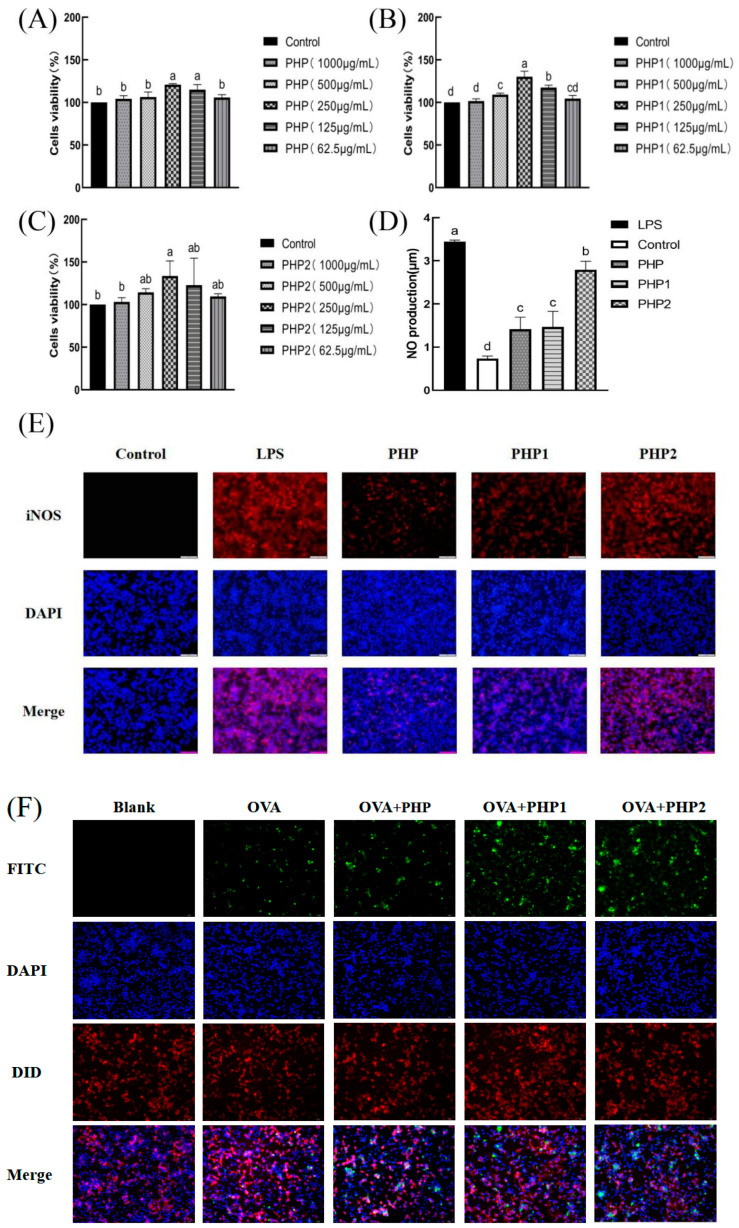

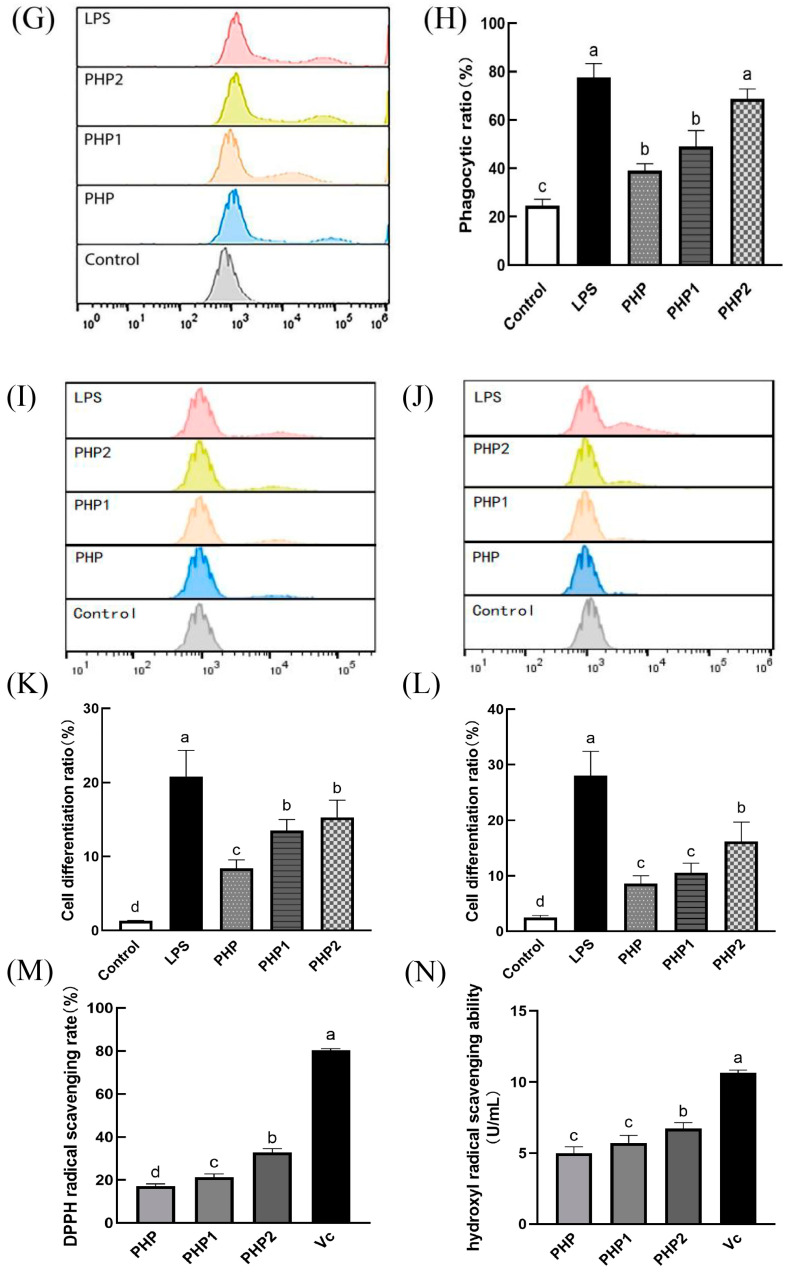
Activity of ultrasonic PHP in RAW264.7. (**A**) Effects of PHP on cell viability; (**B**) effects of PHP1 on cell viability; (**C**) effects of PHP2 on cell viability; (**D**) effects of PHP, PHP1, and PHP2 on NO release from RAW264.7; (**E**) effects of PHP, PHP1, and PHP2 on iNOS expression of RAW264.7; (**F**–**H**) effects of PHP, PHP1, and PHP2 on cell phagocytosis. The expression frequencies of CD80+ (**I**,**K**) and CD86+ (**J**,**L**) in macrophages. (**M**) Effects of PHP, PHP1, and PHP2 on DPPH radical scavenging; (**N**) effects of PHP, PHP1, and PHP2 on hydroxyl radical scavenging. *p* < 0.05 indicates a significant difference. Letters that are not exactly the same indicate significant differences (*p* < 0.05).
