# Supplementary material for: Extraction of Polysaccharides from Root of Pseudostellaria heterophylla (Miq.) Pax. and the Effects of Ultrasound Treatment on Its Properties and Antioxidant and Immune Activities
Source: Molecules. 2023 Dec 26;29(1):142. doi: 10.3390/molecules29010142 (PMC10779800; doi:10.3390/molecules29010142)
Supplement: Supplementary file 1 [file molecules-29-00142-s001.zip › molecules-2766070-supplementary.pdf]

Extraction of Polysaccharides from Pseudollaria Radix and the Effects of Ultrasound Treatment on its Properties and Antioxidant and Immune Activities

Single-factor experiments for the extraction of polysaccharide from *P. heterophylla*

Effect of the number of repeated extractions on the polysaccharide extraction rate

Different extraction times (1, 2, and 3 repeated extractions) were chosen for evaluating the effect of repeated extractions on the extraction rate of polysaccharide from *P. Radix*. The duration of extraction, extraction temperature, and the solid-liquid ratio were kept constant at 1h, 70°C, and 20.0 mL/g, respectively. Figure S1A shown a gradual increase in the extraction rate of polysaccharide with the increased number of repeated extractions. After two extractions of the same sample, polysaccharide were almost completely extracted and the extraction rate reached 3.21%. Third extraction did not change the extraction rate. To simplify subsequent enrichment steps two extraction cycles were taken as optimal.

Effect of the duration of extraction on polysaccharide extraction rate

To evaluate the effect of extraction time on the extraction efficiency of polysaccharide from *P. Radix*, the experiment was conducted for 1, 2, or 3 h. Other variables - temperature, the number of extraction cycles and solid-liquid ratio were kept constant at 70°C, 2 times and 1:15 g/mL, respectively. As shown in Figure S1B, initial increase in the duration of extraction increases the extraction rate of polysaccharide from *P. heterophylla*. Below 80 min, the extraction rate of PHP increased significantly up to 5.79%. Above 80 min, the extraction efficiency of polysaccharide sharply decreased. Therefore, 80 min was the optimal duration of extraction.

Effect of solid-liquid ratio on the extraction efficiency of polysaccharide

The influence of solid-liquid proportion on the extraction rate of polysaccharide from *P. Radix* was evaluated at 1:5, 1:10, 1:15, 1:20 and 1:25 ration (g:mL). The duration of extraction, temperature, and the number of extraction cycles were fixed at 1h, 70°C and 2 times, respectively. As shown in Figure S1C, the larger solvent volume leads to better dissolution of polysaccharide and therefore a higher

extraction rate. The saturated polysaccharide solution is reached at solid-liquid ratio 1:15, with the highest yield of 5.58%. Further increase of solvent volume decreased the polysaccharide yield. Therefore, 1:15 was chosen as the optimum solid-liquid ratio.

Effect of extraction temperature on the extraction rate of polysaccharide

To see how extraction temperature influences the efficiency of polysaccharide extraction, the experiment was performed at 40, 50, 60, 70, and 80°C, keeping the duration of extraction, the number of extraction cycles and the solid-liquid ratio at 80 min, 2 times and 1:15 g/mL, respectively. As can be seen from Figure S1D, the extraction rate of polysaccharide significantly increased when the temperature went from 40 to 50°C, reaching the highest value (5.55%) at 50 °C. Higher temperatures promote thermal diffusion of molecules and the increase dissolution of polysaccharide in water. However, at temperatures above 50°C the extraction efficiency decreased most probably due to thermal degradation of polysaccharide and lost after ethanol treatment. Therefore, 50°C was set as the optimum temperature.

Effect of ethanol concentration on the efficiency of polysaccharide extraction

Different EtOH concentrations (60%, 70%, 80%, 90% and 95%) were chosen in order to investigate its influence on the extraction rate of polysaccharide from *P. Radix*. The other experimental variables - extraction time, temperature, the number of repeated extractions and solid-liquid ratio were kept constant at 80 min, 50°C, 2 cycles and 1:15 g/mL, respectively. Figure S1E shows gradual increase in extraction rate of polysaccharide with the increase in ethanol concentration. At the ethanol concentration of 90%, polysaccharide were almost quantitatively precipitated, the extraction rate reached the highest value, 5.10%. Going to 95% ethanol the extraction rate barely changed. Therefore, 90% ethanol is optimal for the precipitation of polysaccharide.

Results and analysis of orthogonal experiment

An orthogonal experimental design was applied for the optimization of polysaccharide extraction from *P. Radix*, starting from optimum experimental conditions obtained in single factor experiments. Independent variables tested were extraction time (A), solid-liquid ratio (B) and extraction temperature (C). The results of orthogonal test are given in Table S1. Orthogonal range analysis indicated the

greatest influence of extraction temperature on the extraction process, followed by solid-liquid ratio and extraction time. The best combination of variables was A2 B3 C2, with the extraction temperature equal 50°C, solid-liquid ratio 1:15 and 100 min extraction time. A verification experiment was performed, and this combination of experimental conditions yielded 4.85% of polysaccharide.

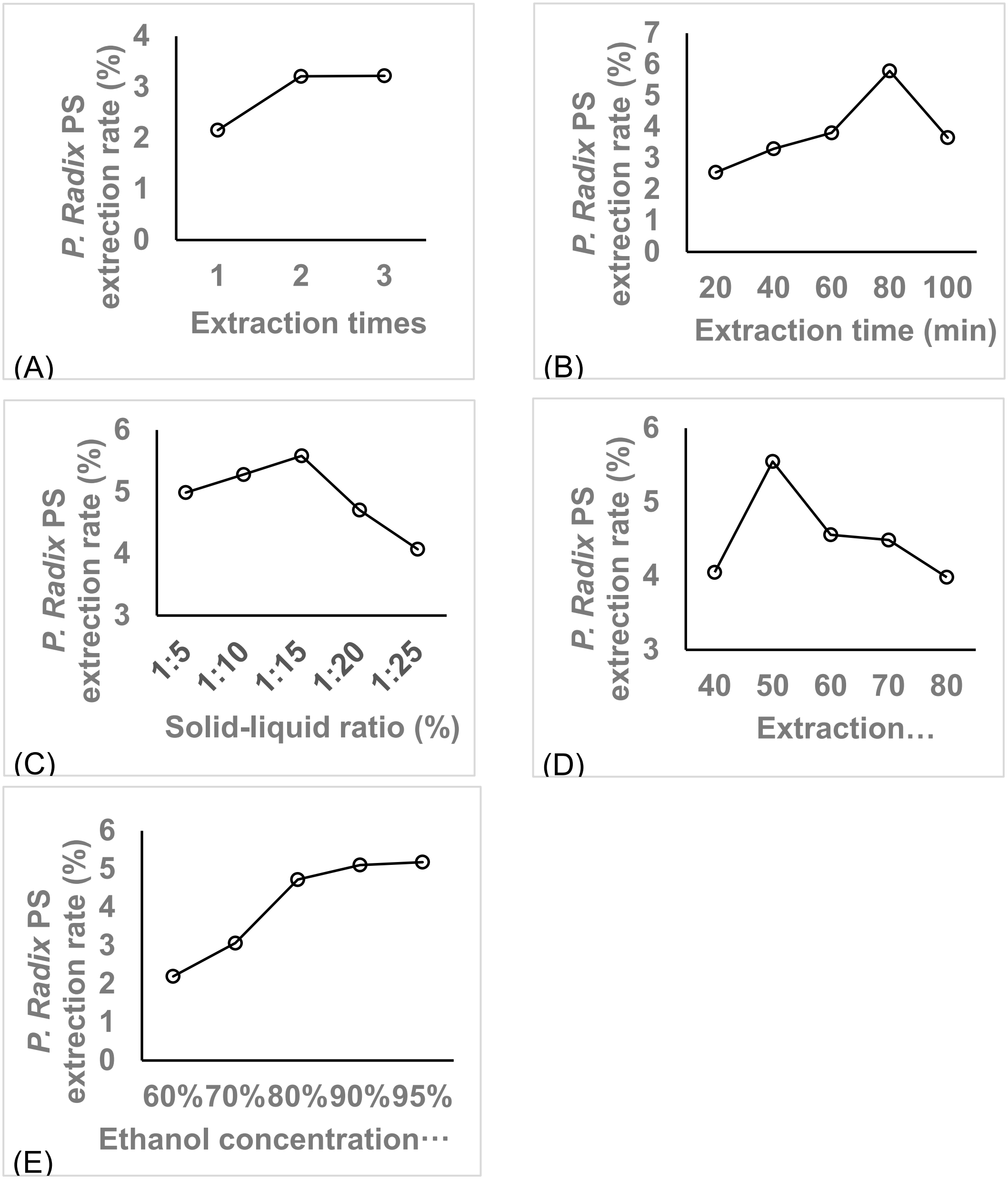

Figure S1 The influence of experimental variables on PHP extraction rate. (A) The number of extraction cycles; (B) Extraction time; (C) Solid-liquid ratio; (D) Temperature; (E) Ethanol concentration.

Table S1 Results and analysis of the orthogonal experiment

Trial	Temperature	Time	Ratio of material to water	Extraction rate
1	1(40)	1(60)	1(1:10)	2.65
2	1	2(80)	2(1:15)	3.95
3	1	3(100)	3(1:20)	3.62
4	2(50)	1	2	5.51
5	2	2	3	5.06
6	2	3	1	5.07
7	3(60)	1	3	4.03
8	3	2	1	3.42
9	3	3	2	4.54
K1	10.22	12.19	11.15	
K2	15.64	12.42	13.99	
K3	11.99	13.23	12.71	
K1 average	3.41	4.06	3.72	
K2 average	5.21	4.14	4.66	
K3 average	4.00	4.41	4.24	
R	1.8	0.35	0.95	
Optimal Level	A2	B3	C2	
